# Mixed competition and technology licensing in a supply chain

**DOI:** 10.3389/fpsyg.2022.966160

**Published:** 2022-09-23

**Authors:** Huaige Zhang, Yu Zhang, Menghuan Zhou

**Affiliations:** ^1^School of Business Administration, Guangdong University of Finance & Economics, Guangzhou, China; ^2^Economics and Management School, Wuhan University, Wuhan, China; ^3^School of Economics and Management, Huazhong Agricultural University, Wuhan, China

**Keywords:** supply chain management, mixed competition, technology licensing, product differentiated, Game Theory

## Abstract

Technology licensing as a vital part of business behavior in many industries has drawn a fair amount of attention in industrial organization literature. Most existing literature on licensing decisions assumes that all firms engage in Cournot or Bertrand competition, while the type of mixed competition may affect the choice of the licensor. In this context, what decision will the licensor make faced with different mixed competitions? This paper studies the optimal technology licensing contract of a licensor firm engaging in different mixed competitions (Cournot-Bertrand or Bertrand-Cournot) with a potential licensee in a differentiated duopoly market considering one upstream firm (supplier) that provides key inputs. We find that if either the royalty or fixed-fee licensing is applied, the licensor favors royalty licensing under Bertrand-Cournot competition when the degree of substitution is small and prefers the fixed-fee licensing no matter under what kind of mixed competition as the degree of substitution increases. In the case of fixed-fee licensing, the result shows that the profits of licensors are the same under different types of mixed competition. Besides, fixed-fee licensing is not always the optimal choice for consumers, and they would prefer royalty licensing when the degree of substitution is very small.

## Introduction

In recent times, as innovation-based competition in business and economic activities become more intense, technology licensing has attracted increasing attention in many industries for its value in innovation development and commercialization (Arora et al., 2013; Agrawal et al., 2016; Hong et al., 2017, 2021; Wu, 2018). Many factors affect the transfer and commercialization of technologies, such as competition mode (Li and Ji, 2010; Nguyen et al., 2014; Zhao et al., 2022); product differentiation (Nguyen et al., 2014, 2017; Bakaouka and Milliou, 2018), imitation costs (Kogan et al., 2013), type of innovation (Agrawal et al., 2016; Chen and Xie, 2018), bargaining power of two parties (Kishimoto, 2020), and network effect (Lin and Kulatilaka, 2006; Zhang et al., 2018). In addition, mixed competition has different influences on market equilibrium as well as on social welfare compared with those under symmetric competition strategies (Tremblay and Tremblay, 2019; Lee et al., 2020; Askar, 2021; Kopel and Putz, 2021). In practice, the Cournot–Bertrand behavior is prevalent in various markets (Tremblay and Tremblay, 2019). In Japan, among the seventy manufacturing industries, thirty industries applied a hybrid price-quantity specification (Flath, 2012). In the automobile industry of Japan, where technology licensing is usually popular, Cournot-Bertrand competition can be found (Tremblay et al., 2013); specifically, Honda and Subaru as quantity setters compete with Scion and Saturn, which are price setters.

However, in the research on technology licensing, much of the literature related to the problems of technology licensing assumes the competition environment is either price competition (Li and Ji, 2010; Nguyen et al., 2014) or quantity competition (Hong et al., 2021; Zhao et al., 2022), and ignore the implications of mixed competition (quantity-price and price-quantity). Chang et al. (2015) investigated an insider innovator's optimal licensing decision issue in the context of mixed competition; however, they ignored the existence of upstream suppliers.

Against this background, this paper extends previous studies on technology licensing in a differentiated duopoly market in which one firm owns an innovative technology and the other is a potential licensee to the case of a different mixed competition considering the role of upstream suppliers. The main purpose is to explore the following three issues. *(1) should the innovator license its technology to the other competitor? (2) what kind of licensing strategy is optimal for the licensor under mixed competition and to what extent should it depend on the degree of substitution? (3) what about the concomitant effects on other participants (consumer surplus, social welfare) in the market?*

To address the above questions, we built a model based on Game Theory where Firm 1 owning a patent for a technology competes with Firm 2 who may be a potential licensee in vertically differentiated products. In this model, if Firm 1 does not license its technology to Firm 2, then the market is a monopoly; since Firm 2 has no technology to produce this product without a license, its products end up as imperfect substitutes. If Firm 1 licenses its technology to Firm 2, then the market is a duopoly. We consider the two types of mixed competition, i.e., the Cournot-Bertrand competition and the Bertrand-Cournot competition.

This study makes the following contributions and conclusions. (1) Compared to the existing licensing studies that have not considered different mixed competitions and upstream suppliers, we find that they play a crucial role in deciding the optimal technology licensing contracts. (2) We find that licensing using royalty under Bertrand-Cournot competition is superior to other modes if the degree of substitution is small, while fixed-fee licensing is optimal for the licensor and consumers no matter under what kind of mixed competition it increases. (3) Conflict occurs between the licensor and supplier when the degree of substitution is limited to 0.17 < *d* < 0.87. Both of their goals are profit maximization, while the supplier would rather Firm 1 not license at that time. (4) Fixed-fee licensing is not always the best choice for consumers when the degree of substitution is very small under mixed competition. (5) Under fixed-fee licensing, the profits of the licensor are the same as under different modes of mixed competition.

The remainder of the paper will proceed as follows. In Section Literature review, we review the related literature. Section Model setup and no-licensing scenario introduces the model and derives the no-licensing status quo as a benchmark. Two licensing strategies (royalty licensing and fixed-fee licensing) are examined respectively in Sections Royalty licensing and Fixed-fee licensing. Section Comparative analysis deals with the optimal licensing strategy and the welfare implications under different conditions. Finally, Section Conclusion concludes the paper.

## Literature review

Early works on technological innovation and technology licensing date back to Arrow (1962), and since then numerous intense and profound studies have followed. The literature on technology licensing of innovative firms mainly includes two key questions: What is the optimal licensing contract? What is the optimal number of licensees? As for the former question, Wang (1998) investigated the fixed-fee and royalty licensing contract for insider licensors in a homogeneous Cournot duopoly market and concluded that royalty licensing is superior for it distorts the marginal cost of the licensee to generate a higher total income. Due to the heterogeneity of the products in reality, Wang (2002) introduced product differentiation extending to a heterogeneous Cournot duopoly market. In contrast, the findings by Kamien and Tauman (1986) showed that fixed-fee licensing was superior to royalty licensing, while Wang (2002) found that royalty licensing was better in most instances. The difference in their finding is determined by whether the patent holder is an insider or not. Some studies examine the impact of other factors such as different market structures (Katz and Shapiro, 1985), type of competition (Kabiraj, 2004), network effect (Lin and Kulatilaka, 2006; Zhang et al., 2018), imitation costs (Kogan et al., 2013), cross-licensing (Zhao, 2017; Jeon and Lefouili, 2018; Choi and Gerlach, 2019; Wang and Huang, 2019; Zhao et al., 2022); and information asymmetry (Sen and Bhattacharya, 2017; Jeon, 2019; Hong et al., 2021).

On the question of the optimal number of licenses, Kamien and Tauman (2002) extended Wang's (1998) to the homogeneous Cournot market and discussed the positive relationship between licensing revenue of the only licensor and the number of firms in the market. Arora and Fosfuri (2003) studied the influence of the licensor's competition on the number of licenses relaxing the assumption of a monopolist technology holder. They show that the number of licenses per patent holder has a negative relationship with the degree of product differentiation. These studies arrive at their conclusions based on the assumption that firms compete in a Cournot quantity model. In this paper, we consider the case where two firms compete in a differentiated duopoly market under mixed competition to explore how the type of mixed competition affects the licensing decision.

The history of oligopoly theory started with Cournot (1838) and Bertrand (1883). Both models derived the non-cooperative equilibrium of Nash (1950). Firms producing differentiated products in a duopoly market simultaneously choose either quantity or price as the strategy variable. Many existing studies focus on the comparison between Cournot and Bertrand's models of competition on the premise of firms formulating the same type of strategy (Naskar and Pal, 2020; Ferreira et al., 2021). Tremblay and Tremblay (2011), Tremblay et al. (2011) found that with homogenous products the quantity-setting firm sets a perfectly competitive level of market output than firms with price setting. Tremblay et al. (2013) reported that the Cournot-Bertrand market competition emerges with the firms' asymmetric fixed costs. Haraguchi and Matsumura (2016) compare Cournot and Bertrand's competition in a mixed oligopoly in which one state-owned public firm competes with private firms. Semenov and Tondji (2019) studied the role of a Cournot-Bertrand market on firms' investment in R&D and found that the firm as a quantity-setter invests more in cost-reduction than the firm as a price-setter. Asproudis and Filippiadis (2021) found that a Cournot firm generally chooses the technology that is more environmental-friendly than a Bertrand firm. Unfortunately, these papers ignore the vertical relationship between the input suppliers and downstream firms under technology licensing, which is quite common in reality. For example, as pointed out in Corbett (2004), more than 90 percent of enterprises regard licensing as a significant business strategy. We apply this insight to explain how upstream suppliers affect the licensing decision of the licensor.

Reviewing the above literature, we find that most papers on insider licensing do not consider the role of upstream suppliers in the context of mixed competition. Arya and Mittendorf (2006) study the choice of optimal technology licensing in a vertically related market with two firms producing a homogeneous product. The licensor has to make a trade-off between competitive and supplier pricing effects. It turns out that all parties including the licensor, the licensee, the supplier, and the consumers can benefit and the presence of an upstream supplier has a significant impact on the decision of licensing strategies. Our analysis extends the above model to a differentiated duopoly under mixed competition. However, this paper differs from previous literature in two aspects. First, we investigate the optimal licensing strategy for the licensor under different mixed competitions. Second, we consider product differentiation in the context of resource outsourcing. Hence, one contribution of this paper is to bring these two factors, namely vertical-related market, and mixed competition, together. In a simple model with heterogeneous final goods, we show that the pricing strategy of the upstream supplier and the competition strategy of the downstream firms play important roles in licensing decisions.

## Model setup and no-licensing scenario

Consider a duopoly model in which Firm 1 owning a patent for a technology competes with Firm 2 who may be a potential licensee in vertically differentiated products. If licensing does not occur, Firm 1 becomes a monopolist. For simplicity, we assume that Firm 2 has no technology to produce this product without a license, and the products of Firm 2 are imperfect substitutes. Besides, they both need a supplier to provide the intermediate good, one unit of which takes a constant cost *c*and we normalize the firms' marginal cost of input production to zero. We adopt a standard differentiated duopoly with a linear demand (Dixit, 1979; Wang, 2002; Agrawal et al., 2016; Chen et al., 2017), where the linear inverse demand functions for Firms 1 and 2 are *p*_1_(*q*_1_, *q*_2_) = *a* − *q*_1_ − *dq*_2_ and *p*_2_(*q*_1_, *q*_2_) = *a* − *q*_2_ − *dq*_1_, respectively. While *a* is the maximum price that consumers will pay for one unit of vertically differentiated product and *a* > *c*; *p*_*i*_ is Firm *i*'s price and *q*_*i*_ is Firm *i*'s quantity (*i* = 1, 2). The coefficient *d* captures the degree of substitution between the two kinds of products. The larger the *d*, the fewer the differences there are between the products, *d* ∈ [0, 1]. If *d* = 0, the demands are independent; if *d* = 1, the products are perfect substitutes.

We can obtain the demand functions of the two firms regarding price as $q_{1}\left(p_{1},p_{2}\right)\;=\;\frac{a\left(1\;-\;d\right)\;+\;dp_{2}\;-\;p_{1}}{1\;-\;d^{2}}$ and $q_{2}\left(p_{1},p_{2}\right)\;=\;\frac{a\left(1\;-\;d\right)\;+\;dp_{1}\;-\;p_{2}}{1\;-\;d^{2}}$, which are downward-sloping in their own prices and increasing in the rival's price functions when the goods are substitutes. Under Cournot-Bertrand competition, the demand functions are $p_{1}\;=\;a\left(1\;-\;d\right)\;-\;{\left(1\;-\;d\right)}^{2}q_{1}\;+\;dp_{2}$ and *q*_2_ = *a* − *p*_2_ − *dq*_1_. In terms of Bertrand-Cournot competition, they are *q*_1_ = *a* − *p*_1_ − *dq*_2_ and $p_{2}\;=\;a\left(1\;-\;d\right)\;-\;{\left(1\;-\;d\right)}^{2}q_{2}\;+\;dp_{1}$.

Throughout this paper, we label firm *i*'s profit as $\pi_{ij}^{kl}$ to make it easy to distinguish under mixed competition, where N denotes no licensing, F indicates a fixed fee and R means a royalty. $CS_{j}^{kl}$ and $W_{j}^{kl}$ represent the corresponding consumer surplus and social welfare. For example, $\pi_{1N}^{CB}$ means Firm 1's profit when licensing does not occur under Cournot-Bertrand competition, and $CS_{N}^{kl}$ is the consumer surplus under this situation.

We first consider a Cournot monopoly that licensing does not occur as a benchmark. Meaning that Firm 1 becomes the monopolist. Following the above model, we can get the equilibrium by using backward induction. The demand function becomes *p* = *a* − *q*_1_. Suppose a supplier charges a price *w*_1_, Firm 1 will choose the optimal quantity to maximize its profit.


(1) $$\begin{array}{l} \underset{q_{1}}{Max}\left(a-q_{1}\right)q_{1}\;-\;w_{1}q_{1}. \end{array}$$

The first-order condition of (1) with respect to *q*_1_ yields:


(2) $$\begin{array}{l} q_{1}\;=\;\left(a\;-\;w_{1}\right)/2. \end{array}$$

Therefore, the supplier chooses *w*_1_ to maximize its profit,


(3) $$\begin{array}{l} \underset{w_{1}}{Max}\left(w_{1}-c\right)q_{1N}^{CB}. \end{array}$$

When the price *w*_1_ is settled, the optimal price for the supplier to charge in the no-licensing scenario, substituting $w_{1N}^{CB}$ with (2) gives:


(4) $$\begin{array}{l} w_{1N}^{CB}\;=\;\frac{\left(a\;+\;c\right)}{2}\text{ and }q_{1N}^{CB}\;=\;\frac{a\;-\;c}{4}. \end{array}$$

In this case, we obtain the equilibrium profits of Firm 1 and the supplier:


(5) $$\begin{array}{l} \pi_{1N}^{CB}\;=\;\frac{{\left(a-c\right)}^{2}}{16}\text{ and }\pi_{SN}^{CB}\;=\;\frac{{\left(a-c\right)}^{2}}{8}. \end{array}$$

Consumer surplus equals:


(6) $$\begin{array}{l} CS_{N}^{CB}\;=\;\int_{0}^{q_{1N}^{CB}}\left(q_{1N}^{CB}-q\right)dq\;=\;\frac{1}{2}{\left(q_{1N}^{CB}\right)}^{2}\;=\;\frac{{\left(a-c\right)}^{2}}{32}. \end{array}$$

and social welfare:


(7) $$\begin{array}{ll} W_{N}^{CB}\;=\;\pi_{1N}^{CB}\;+\;\pi_{SN}^{CB}+CS_{N}^{CB}\;=\;\frac{{\left(a\;-\;c\right)}^{2}}{16}\;+\;\frac{{\left(a-c\right)}^{2}}{8}\;\\ \;+ & \frac{{\left(a\;-\;c\right)}^{2}}{32}\;=\;\frac{7{\left(a\;-\;c\right)}^{2}}{32}. \end{array}$$

When Firm 1 chooses the optimal price instead of quantity to maximize its profit, the status quo is the same as quantity competition,


(8) $$\begin{array}{l} \underset{p_{1}}{Max}\left(a\;-\;p_{1}\right)\left(p_{1}\;-\;w_{1}\right). \end{array}$$

The fist-order condition of (8) in terms of *p*_1_ yields the firm's optimal price and supply of the product in the no-license scenario:


(9) $$\begin{array}{l} p_{1N}^{BC}\;=\;\frac{a\;+\;w_{1}}{2},\;q_{1N}^{BC}\;=\;\frac{a\;-\;w_{1}}{2}. \end{array}$$

Actually, (9) serves as the induced demand function for the supplier who chooses*w*_1_,


(10) $$\begin{array}{l} Max\left(w_{1}\;-\;c\right)q_{1}. \end{array}$$

Similarly, we get


(11) $$\begin{array}{l} w_{1N}^{BC}\;=\;\frac{a+c}{2}\text{ and }q_{1N}^{BC}\;=\;\frac{a-c}{4}. \end{array}$$

As we can see, regardless of what strategy Firm 1 adopts, price or quantity, the supplier charges the same and higher than the marginal cost under the no-licensing scenario. Firm 1 chooses the same optimal quantity but less than it would have when the supplier charges *c*. This is the recognized double marginalization problem in supply chains. The total profits are less than if the supply chain members were vertically integrated. Then the profits and responding consumer surplus and social welfare are


(12) $$\begin{array}{l} \pi_{1N}^{BC}\;=\;\frac{{\left(a-c\right)}^{2}}{16},\pi_{SN}^{BC}\;=\;\frac{{\left(a-c\right)}^{2}}{8}, \end{array}$$

(13) $$\begin{array}{l} CS_{N}^{BC}\;=\;\int_{0}^{q_{1N}^{BC}}\left(q_{1N}^{BC}\;-\;q\right)dq=\frac{1}{2}{\left(q_{1N}^{BC}\right)}^{2}=\frac{{\left(a-c\right)}^{2}}{32}, \end{array}$$

(14) $$\begin{array}{l} W_{N}^{BC}\;=\;\pi_{1N}^{BC}\;+\;\pi_{SN}^{BC}\;+\;CS_{N}^{BC}\;=\;\frac{7{\left(a\;-\;c\right)}^{2}}{32}. \end{array}$$

## Royalty licensing

### Cournot-Bertrand competition

In this part, the royalty licensing in the Cournot-Bertrand competition and the Bertrand-Cournot competitions are discussed respectively. Finally, we compare the two alternative licensing contracts under different mixed competitions. By issuing the license, Firm 1 loses its monopoly position. Once a licensing agreement is reached, Firm 2 gets the chance to produce different goods in the market.

It appears that Firm 1 would lose part of the market share and in actuality, it is a trade-off for the licensor. Patent licensing will lead to two kinds of effects: On the one hand, the innovative firm can extract some licensing revenues from the licensee, that is, the licensing effect; on the other hand, it will incur a loss because of the licensee's competitiveness, that is, the competition effect. When the licensing effect exceeds the competitive effect, technology licensing is beneficial to the innovation side.

The licensing game is simulated as follows: First, the supplier decides prices *w*_1_ and *w*_2_. Second, Firm 1 decides to license its cost-reducing innovation to Firm 2 or not by making a take-it-or-leave-it offer. Then Firm 2 accepts or refuses it. Finally, the two firms choose their own optimal quantity or price under mixed competition simultaneously. The game is again solved by backward induction given a royalty rate *r* per unit of output and supplier prices *w*_1_ and *w*_2_. Using the model mentioned before, when Firm 1 chooses *q*_1_ to maximize,


(15) $$\begin{array}{ll} \underset{q_{1}}{Max}\pi_{1}\;=\;\left[a\left(1\;-\;d\right)\;-\;\left(1\;-\;d^{2}\right)q_{1}\;+\;dp_{2}\right]q_{1}\\ \;- & \;w_{1}q_{1}\;+\;r\left(a\;-\;p_{2}-dq_{1}\right). \end{array}$$

and yields Firm 1's quantity-reaction function


(16) $$\begin{array}{l} q_{1}\;=\;\frac{a\left(1\;-\;d\right)\;+\;dp_{2}\;-\;w_{1}\;-\;dr}{2\left(1\;-\;d^{2}\right)}. \end{array}$$

Firm 2's price-reaction function is obtained by:


(17) $$\begin{array}{l} \underset{p_{2}}{Max}\pi_{2}\;=\;\left(a\;-\;p_{2}\;-\;dq_{1}\right)\left(p_{2}\;-\;w_{2}\;-\;r\right). \end{array}$$

Using the first-order condition of (17), we get:


(18) $$\begin{array}{l} p_{2}\;=\;\frac{a\;-\;dq_{1}\;+\;w_{2}\;+\;r}{2}. \end{array}$$

In (18), Firm 2 effectively internalizes the royalty rate as an added cost of production, higher royalty leads to a higher price. Assuming an internal solution, the intersection of these reaction functions yields the firms' Cournot-Bertrand equilibrium quantities:


(19) $$\begin{array}{l} q_{1}\;=\;\frac{2a\;-\;ad\;-\;dr\;-\;2w_{1}\;+\;dw_{2}}{4\;-\;3d^{2}},\\ \;q_{2}\;=\;\frac{2a-ad^{2}-ad+dw_{1}-\left(2-d^{2}\right)w_{2}-\left(2-2d^{2}\right)r}{4-3d^{2}},\\ \;p_{2}=\frac{2a-ad^{2}-ad+dw_{1}+\left(2-2d^{2}\right)w_{2}+\left(2-d^{2}\right)r}{4-3d^{2}}. \end{array}$$

Not surprisingly, each firm's quantity is decreasing in the price it pays to the supplier; part of Firm 2's price is related to Firm 1's cost for intermediate goods, and a higher price for the competitor stimulates Firm 1 to increase its production. The supplier-set prices are determined by:


(20) $$\begin{array}{l} \underset{w_{1},w_{2}}{Max}\pi_{s}\;=\;\left(w_{1}\;-\;c\right)q_{1}\;+\;\left(w_{2}\;-\;c\right)q_{2}. \end{array}$$

Substituting *q*_1_ and *q*_2_with (20), the first-order condition of (20) yields the outcomes:


(21) $$\begin{array}{l} w_{1}\;=\;\frac{2a-ad-dr+2dw_{2}+2c-cd}{4},\\ \;w_{2}=\frac{\left(2-d-d^{2}\right)a-\left(2-2d^{2}\right)r+2dw_{1}+\left(2-d^{2}-d\right)c}{\left(2-2d^{2}\right)}. \end{array}$$

And the profits of the two firms

Similarly, the intersections of these outcomes determine the prices with licensing:


(22) $$\begin{array}{l} w_{1R}^{CB}\;=\;\frac{a\;+\;c\;-\;dr}{2}\text{ and }w_{2R}^{CB}\;=\;\frac{a\;+\;c\;-\;r}{2}. \end{array}$$

The price offered to Firm 1 is lower than before due to the royalty rate and the degree of differentiation but is higher than that of Firm 2. As we know, Firm 1 licenses to Firm 2 at a royalty rate and extracts a part of the profit from this. Firm 2 is less competitive so the supplier sets a lower price to maintain the demand. The result shows that to procure a better price from the supplier, Firm 1 may choose to provide the license to a competitor.

Substituting (22) with (19), we obtain the equilibrium quantities and prices in terms of royalty rate as follows:


(23) $$\begin{array}{l} q_{1R}^{CB}\;=\;\frac{\left(2\;-\;d\right)\left(a\;-\;c\right)\;-\;dr}{2\left(4\;-\;3d^{2}\right)},\\ \;q_{2R}^{CB}\;=\;\frac{\left(2\;-\;d-d^{2}\right)\left(a\;-\;c\right)\;+\;\left(2d^{2}\;-\;2\right)r}{2\left(4\;-\;3d^{2}\right)},\\ \;p_{1R}^{CB}\;=\;\frac{\begin{array}{c} \left(6\;-\;d\;-\;5d^{2}\;+\;d^{3}\right)a\;+\;\left(2\;+\;d\;-\;d^{2}\;-\;d^{3}\right)c\;+\\ \;\left(3d\;-\;2d^{3}\right)r \end{array}}{2\left(4\;-\;3d^{2}\right)},\\ \;p_{2R}^{CB}=\frac{\left(6-d-4d^{2}\right)a+\left(2-2d^{2}+d\right)c+\left(2-d^{2}\right)r}{2\left(4-3d^{2}\right)}. \end{array}$$

Note that if $r=\frac{\left(2-d-d^{2}\right)\left(a-c\right)}{2-2d^{2}}$, then $q_{2R}^{CB}=0$ and $q_{1R}^{CB}=\frac{a-c}{4\left(1+d\right)}$, which means there is only Firm 1 in the market, and *d* is supposed to be zero at this time, and $q_{1R}^{CB}$ is similar to the monopoly outcome in a no-licensing scenario. The maximum royalty rate Firm 1 sets cannot exceed $r^{*}=\frac{\left(2-d-d^{2}\right)\left(a-c\right)}{2-2d^{2}}$. Substituting (22) and (23) with (15) provides Firm 1's profit and royalty rate accordingly.


(24) $$\begin{array}{l} \underset{r}{Max\pi_{1R}^{CB}}\;=\;\left(p_{1}\;-w_{1}\right)q_{1}\;+\;rq_{2}. \end{array}$$

The first-order condition of (24) with respect to *r* provides the royalty rate:


(25) $$\begin{array}{l} r_{1}^{CB}\;=\;\frac{\left(5d^{4}\;-\;d^{3}\;-\;13d^{2}\;+\;2d\;+\;8\right)\left(a\;-\;c\right)}{7d^{4}\;-\;21d^{2}\;+\;16}. \end{array}$$

The maximum royalty rate that Firm 2 agrees to is under constraint $\pi_{2R}^{CB}\;-\;\pi_{2N}^{CB}\;=\;\left(p_{2}\;-\;w_{2}\;-\;r\right)q_{2}\;=\;\frac{{\left[\left(2\;-\;d\;-\;d^{2}\right)\left(a\;-\;c\right)\;-\;\left(2\;-\;2d^{2}\right)r\right]}^{2}}{4{\left(4\;-\;3d^{2}\right)}^{2}}\;=\;0$, and the result is $r_{{\;}_{2}}^{CB}\;=\;\frac{\left(2\;-\;d\;-\;d^{2}\right)\left(a\;-\;c\right)}{2\;-\;2d^{2}}\;=\;r^{*}$.

Since the profit of Firm 2 is zero when licensing does not occur, as long as the royalty rate is no more than r^*^, the situation of Firm 2 will always be better so it will accept the license. In fact, $r_{{\;}_{1}}^{CB}<r_{2}^{CB}$ holds for all *d* ∈ (0, 1).

Substituting with (23) yields:


(26) $$\begin{array}{l} q_{1R}^{CB}\;=\;\frac{\left(-\;12d^{5}\;+\;15d^{4}\;+\;34d^{3}\;-\;44d^{2}\;-\;24d\;+\;32\right)\left(a\;-\;c\right)}{2\left(7d^{4}\;-\;21d^{2}\;+\;16\right)\left(4\;-\;3d^{2}\right)}\text{ and}\\ \;q_{2R}^{CB}=\frac{\left(3d^{6}-9d^{5}\;-\;d^{4}\;+\;27d^{3}\;-\;16d^{2}\;-\;20d\;+\;16\right)\left(a\;-\;c\right)}{2\left(7d^{4}\;-\;21d^{2}+16\right)\left(4-3d^{2}\right)}. \end{array}$$

And the profits of the two firms and the supplier can be obtained:


$$\begin{array}{l} \pi_{1R}^{CB}=\frac{\begin{array}{c} (126d^{12}+126d^{11}-1\text{281}d^{10}-840d^{9}+5237d^{8}\\ +2234d^{7}-11136d^{6}-2960d^{5}+13072d^{4}\\ +1952d^{3}-8064d^{2}-512d+2048){\left(a-c\right)}^{2} \end{array}}{4{\left(4-3d^{2}\right)}^{2}{\left(7d^{4}-21d^{2}+16\right)}^{2}}, \end{array}$$

$$\begin{array}{l} \pi_{2R}^{CB}\;=\;\frac{{\left(3d^{6}\;-\;9d^{5}\;-\;d^{4}\;+\;27d^{3}\;-\;16d^{2}\;-\;20d\;+\;16\right)}^{2}{\left(a\;-\;c\right)}^{2}}{4{\left(4\;-\;3d^{2}\right)}^{2}{\left(7d^{4}\;-\;21d^{2}\;+\;16\right)}^{2}}, \end{array}$$

(27) $$\begin{array}{l} \pi_{SR}^{CB}=\frac{\begin{array}{c} (66d^{10}-186d^{9}-241d^{8}+1082d^{7}+6d^{6}-\\ 2360d^{5}+1022d^{4}+2288d^{3}-1464d^{2}-832d\\ +640){\left(a-c\right)}^{2} \end{array}}{4(4-3d^{2}){\left(7d^{4}-21d^{2}+16\right)}^{2}}. \end{array}$$

Only when $\pi_{1R}^{CB}\geq \pi_{1N}^{CB}$, can royalty licensing occur. The following Proposition 1a deals with the statement by comparing the results of royalty licensing and no-licensing.

**Proposition 1a:** *Compared with the no-licensing status quo, royalty licensing under Cournot-Bertrand competition is strictly profitable for the licensor*.

It is easy to understand that Firm 2 benefits from procuring the license as it accesses a new market. The fact that satisfaction with the licensing arrangement drags down the profit of the supplier in most cases is more of a surprise. Unless the degree of substitution is small enough, the supplier would not benefit from licensing. Comparing the supplier's profit in (27) and (12) verifies this. The reason is that issuing a license can influence the supplier's behavior. Licensing creates competition between the two firms, and such competition yields higher demand than in the no licensing scenario. However, there is an offsetting factor that outweighs the benefits for the supplier—lower price.

When licensing occurs under a royalty contract in Cournot-Bertrand competition, the responding consumer surplus and social welfare are:


$$\begin{array}{l} CS_{R}^{CB}=[a(q_{1}+q_{2})-\frac{q_{1}^{2}+2q_{1}q_{2}+q_{2}^{2}}{2}-p_{1}q_{1}-p_{2}q_{2}]\\ \;=\frac{\begin{array}{c} (-45d^{12}+180d^{11}+483d^{10}-2850d^{9}\\ +798d^{8}+10278d^{7}-7353d^{6}-17352d^{5}\\ +15720d^{4}+12960d^{3}-13200d^{2}-3456d+\\ 3840){\left(a-c\right)}^{2} \end{array}}{8{\left(4-3d^{2}\right)}^{2}{\left(7d^{4}-21d^{2}+16\right)}^{2}}, \end{array}$$

(28) $$\begin{array}{l} W_{R}^{CB}\;=\;\pi_{1R}^{CB}\;+\;\pi_{2R}^{CB}\;+\;\pi_{SR}^{CB}\;+\;CS_{R}^{CB}. \end{array}$$

Comparing consumer surplus in (28) and (13), we can see that the former is strictly larger, and the increased effect outweighs the decreased benefits as social welfare increases. These comparisons confirm the following proposition.

**Proposition 1b:** *Firm 1's decision to transfer technology by royalty licensing benefits Firm 2, consumers, and the whole society while the supplier gets less profit in most instances in Cournot-Bertrand competition*.

Proposition 1a and 1b imply that if Firm 1 adopts a quantity strategy while Firm 2 chooses a price strategy, the former will transfer the technology by royalty licensing. The result shows that all the participants in the market except the supplier strictly get benefits. As mentioned above, the supplier is compelled to lower prices for the competitor's sake, so the patent holder again gets benefits apart from the gains from the royalty fee. Although the total market demand increases, the net effect indicates that the supplier has a loss if the degree of substitution exceeds 0.17. This is the critical point of the two - fold effect for the supplier since the price offered to Firm 1 decreases as the degree of substitution increases. In addition, royalty licensing can reduce the double marginalization problem of the supply chain.

### Bertrand-Cournot competition

The outcomes are different when analyzing through the Bertrand-Cournot competition. When Firm 1 chooses the right price to maximize its profit,


(29) $$\begin{array}{ll} \underset{p_{1}}{Max}\pi_{1}\;=\;p_{1}\left(a\;-\;p_{1}\;-\;dq_{2}\right)\;-\;w_{1}\left(a\;-\;p_{1}\;-\;dq_{2}\right)\\ \;+ & \;r\frac{a\left(1\;-\;d\right)\;-\;p_{2}\;+\;dp_{1}}{1\;-\;d^{2}}. \end{array}$$

the first order condition of (29) with regard to*p*_1_yields the price - reaction function as


(30) $$\begin{array}{l} p_{1}\;=\;\frac{a\;-\;dq_{2}\;+\;w_{1}}{2}\;+\;\frac{dr}{2\left(1\;-\;d^{2}\right)}. \end{array}$$

Firm 2's response function is obtained by:


(31) $$\begin{array}{ll} \underset{q_{2}}{Max}\pi_{2}\;=\;\left(a\left(1\;-\;d\right)\;-\;\left(1\;-\;d^{2}\right)q_{2}\;+\;dp_{1}\right)q_{2}\\ \;- & \;w_{2}q_{2}\;-\;rq_{2}. \end{array}$$

And it yields the price-reaction function as:


(32) $$\begin{array}{l} q_{2}\;=\;\frac{a\left(1\;-\;d\right)\;+\;dp_{1}\;-\;w_{2}\;-\;r}{2\left(1\;-\;d^{2}\right)}. \end{array}$$

Assuming an internal solution, the intersection of these reaction functions yields the firms' Bertrand-Cournot equilibrium quantities:


$$\begin{array}{l} p_{1}\;=\;\frac{\left(2\;-\;d\;-\;d^{2}\right)a\;+\;2\left(1\;-\;d^{2}\right)w_{1}\;+\;dw_{2}\;+\;3dr}{4\;-\;3d^{2}}, \end{array}$$

$$\begin{array}{ll} q_{1}\;=\;\frac{\left(2\;-\;d\;-\;d^{2}\right)a\;+\;\left(2\;-\;d^{2}\right)w_{1}\;+\;dw_{2}}{4\;-\;3d^{2}}\;\\ \;- & \frac{dr}{\left(4\;-\;3d^{2}\right)\left(1\;-\;d^{2}\right)}, \end{array}$$

(33) $$\begin{array}{l} q_{2}=\frac{\left(2-d\right)a+dw_{1}-2w_{2}}{4-3d^{2}}+\frac{\left(3d^{2}-2\right)r}{\left(4-3d^{2}\right)\left(1-d^{2}\right)}. \end{array}$$

The supplier-set prices are determined by:


(34) $$\begin{array}{l} \underset{w_{1},w_{2}}{Max}\pi_{s}\;=\;\left(w_{1}\;-\;c\right)q_{1}\;+\;\left(w_{2}\;-\;c\right)q_{2}. \end{array}$$

When *q*_1_ and *q*_2_ are substituted with (34), the first - order condition of (34) yields the outcomes:


(35) $$\begin{array}{l} w_{1}=\frac{\left(2-d\;-\;d^{2}\right)\left(a\;+\;c\right)\;+\;2dw_{2}}{2\left(2\;-\;d^{2}\right)}-\frac{dr}{2\left(2\;-\;d^{2}\right)\left(1\;-\;d^{2}\right)},\\ \;w_{2}\;=\;\frac{\left(2\;-\;d\right)\left(a\;+\;c\right)\;+\;2dw_{1}\;-\;2r\;+\;\frac{d^{2}r}{1\;-\;d^{2}}}{4}. \end{array}$$

In a similar fashion, the intersection of these outcomes proves the prices under the licensing scenario:


(36) $$\begin{array}{l} w_{1R}^{BC}\;=\;\frac{a\;+\;c}{2}\;-\;\frac{dr}{2\left(1\;-\;d^{2}\right)},w_{{\;}_{2R}}^{BC}\;=\;\frac{a\;+\;c\;-\;r}{2}. \end{array}$$

The price offered to Firm 1 is lower than before due to the royalty rate and the degree of differentiation being lower in the Cournot-Bertrand competition. When 0.618 < *d* < 1, the price offered to Firm 1 is lower than the price offered to Firm 2. It appears that the competition regimes have no influence on the price offered to Firm 2. Substituting (36) with (33), the equilibrium quantities and prices are as follows:


(37) $$\begin{array}{l} q_{1R}^{BC}\;=\;\frac{\left(2\;-\;d\;-\;d^{2}\right)\left(a\;-\;c\right)}{2\left(4\;-\;3d^{2}\right)}\;-\;\frac{dr}{2\left(4\;-\;3d^{2}\right)\left(1\;-\;d^{2}\right)},\\ \;q_{2R}^{BC}\;=\;\frac{\left(2\;-\;d\right)\left(a\;-\;c\right)}{2\left(4\;-\;3d^{2}\right)}\;+\;\frac{\left(3d^{2}\;-\;2\right)r}{2\left(4\;-\;3d^{2}\right)\left(1\;-\;d^{2}\right)},\\ \;p_{1R}^{BC}\;=\;\frac{\left(6\;-\;d\;-\;4d^{2}\right)a\;+\;\left(2\;+\;d\;-\;2d^{2}\right)c\;+\;3dr}{2\left(4\;-\;3d^{2}\right)},\\ \;p_{2R}^{BC}=\frac{\left(6-d-5d^{2}+d^{3}\right)a+\left(2+d-d^{2}-d^{3}\right)c+2r}{2\left(4-3d^{2}\right)}. \end{array}$$

When $r_{{\;}_{2}}^{BC}\;=\;\frac{\left(2\;-\;d\right)\left(1\;-\;d^{2}\right)\left(a\;-\;c\right)}{2\;-\;3d^{2}}$, then $q_{2R}^{BC}\;=\;0$ and $q_{1R}^{BC}\;=\;\frac{\left(3d^{4}\;-\;3d^{3}\;-\;7d^{2}\;-\;4d\;+\;4\right)\left(a\;-\;c\right)}{2\left(4\;-\;3d^{2}\right)\left(2\;-\;3d^{2}\right)}$, which means there is only Firm 1 in the market and *d* is supposed to be zero, the quantity is the same as in a monopoly situation in a no - licensing scenario. And the royalty rate that Firm 1 sets can't exceed $r*\;=\;\frac{\left(2\;-\;d\right)\left(1\;-\;d^{2}\right)\left(a\;-\;c\right)}{2\;-\;3d^{2}}$. Substituting (36) and (37) with (29) provides Firm 1's profit and royalty rate:


(38) $$\begin{array}{l} \underset{r}{Max}\pi_{1R}^{BC}\;=\;\left(p_{1}\;-\;w_{1}\right)q_{1}\;+\;rq_{2}. \end{array}$$

The first-order condition of (38) with respect to *r* yields the royalty rate:


(39) $$\begin{array}{l} r_{{\;}_{1}}^{BC}\;=\;\frac{{\left(1\;-\;d^{2}\right)}^{2}\left(9d^{2}\;-\;2d\;-\;8\right)\left(a\;-\;c\right)}{18d^{6}\;-\;48d^{4}\;+\;45d^{2}\;-\;16}. \end{array}$$

The maximum royalty rate that Firm 2 agrees with is under the constraint $\pi_{2R}^{BC}\;-\;\pi_{2N}^{BC}\;=\;\frac{\left[\left(2\;-\;d\;-\;2d^{2}\;+\;d^{3}\right)\left(a\;-\;c\right)\;+\;\left(3d^{2}\;-\;2\right)r\right]\left[\left(2\;-\;d\right)\left(a\;-\;c\right)\;+\;\frac{\left(3d^{2}\;-\;2\right)r}{1\;-\;d^{2}}\right]}{4{\left(4\;-\;3d^{2}\right)}^{2}}\;=\;0$,

and the result is $r_{{\;}_{2}}^{BC}\;=\;\frac{\left(2\;-\;d\right)\left(1\;-\;d^{2}\right)\left(a\;-\;c\right)}{2\;-\;3d^{2}}\;=\;r^{*}$.

Since the profit of Firm 2 is zero when licensing does not occur, as long as the royalty rate is no more than r^*^, the situation of Firm 2 will always be better and it will accept the license. In fact, $r_{{\;}_{1}}^{BC}<r_{2}^{BC}$ holds for all *d* ∈ (0, 1).

Substituting $r_{1}^{BC}$ with (39) yields:


$$q_{1R}^{BC}=\frac{\begin{array}{c} (-18d^{8}-18d^{7}+84d^{6}+57d^{5}-143d^{4}-62d^{3}\\ +108d^{2}+24d-32)(a-c) \end{array}}{2(18d^{6}-48d^{4}+45d^{2}-16)(4-3d^{2})},$$

(40) $$\begin{array}{l} q_{1R}^{BC}=\frac{\begin{array}{c} (-18d^{7}+9d^{7}+54d^{5}-27d^{4}-55d^{3}+32d^{2}\\ +20d-16)(a-c) \end{array}}{2(18d^{6}-48d^{4}+45d^{2}-16)(4-3d^{2})}. \end{array}$$

And the profits of the two firms and the supplier can be obtained:


$$\begin{array}{l} \pi_{1R}^{BC}=\frac{\begin{array}{c} (324d^{16}+648d^{15}-4\text{158}d^{14}-4140d^{13}+\\ 19728d^{12}+11160d^{11}-49371d^{10}-17076d^{9}\\ +74055d^{8}+16074d^{7}-69680d^{6}-9424d^{5}+\\ 40752d^{4}+3232d^{3}-13696d^{2}-512d+\\ 2048){\left(a-c\right)}^{2} \end{array}}{4{\left(4-3d^{2}\right)}^{2}{\left(18d^{6}-48d^{4}+45d^{2}-16\right)}^{2}}, \end{array}$$

$$\pi_{2R}^{BC}=\frac{\begin{array}{c} (-324d^{16}+324d^{15}+2184d^{14}-2268d^{13}\\ -6273d^{12}+7002d^{11}+9765d^{10}-12420d^{9}\\ -8524d^{8}+13690d^{7}+3481d^{6}-9368d^{5}\\ +312d^{4}+3680d^{3}-880d^{2}-640d+256{)}^{2}\\ {\left(a-c\right)}^{2} \end{array}}{4{\left(4-3d^{2}\right)}^{2}{\left(18d^{6}-48d^{4}+45d^{2}-16\right)}^{2}},$$

$$\pi_{SR}^{CB}=\frac{\begin{array}{c} (-324d^{14}-648d^{13}+2295d^{12}+3888d^{11}\\ -7062d^{10}-9804d^{9}-12358d^{8}+13378d^{7}\\ -3407d^{6}-10480d^{5}+9078d^{4}+4496d^{3}\\ -3576d^{2}-832d+640){\left(a-c\right)}^{2} \end{array}}{4(4-3d^{2}){\left(18d^{6}-48d^{4}+45d^{2}-16\right)}^{2}}.$$

To make royalty licensing possible, $\pi_{1N}^{BC}\;-\;\pi_{1N}^{BC}>0$ has to be satisfied, which needs 0 ≤ *d* ≤ 0.81 holding.

**Proposition 2a:** *Compared with the no-licensing status quo, royalty licensing in Bertrand-Cournot competition is preferable for the licensor when* 0 ≤ *d* ≤ 0.81 *holding, Firm 1 can hardly license if the degree of substitution gets larger than 0.81*.

**Figure 1:**
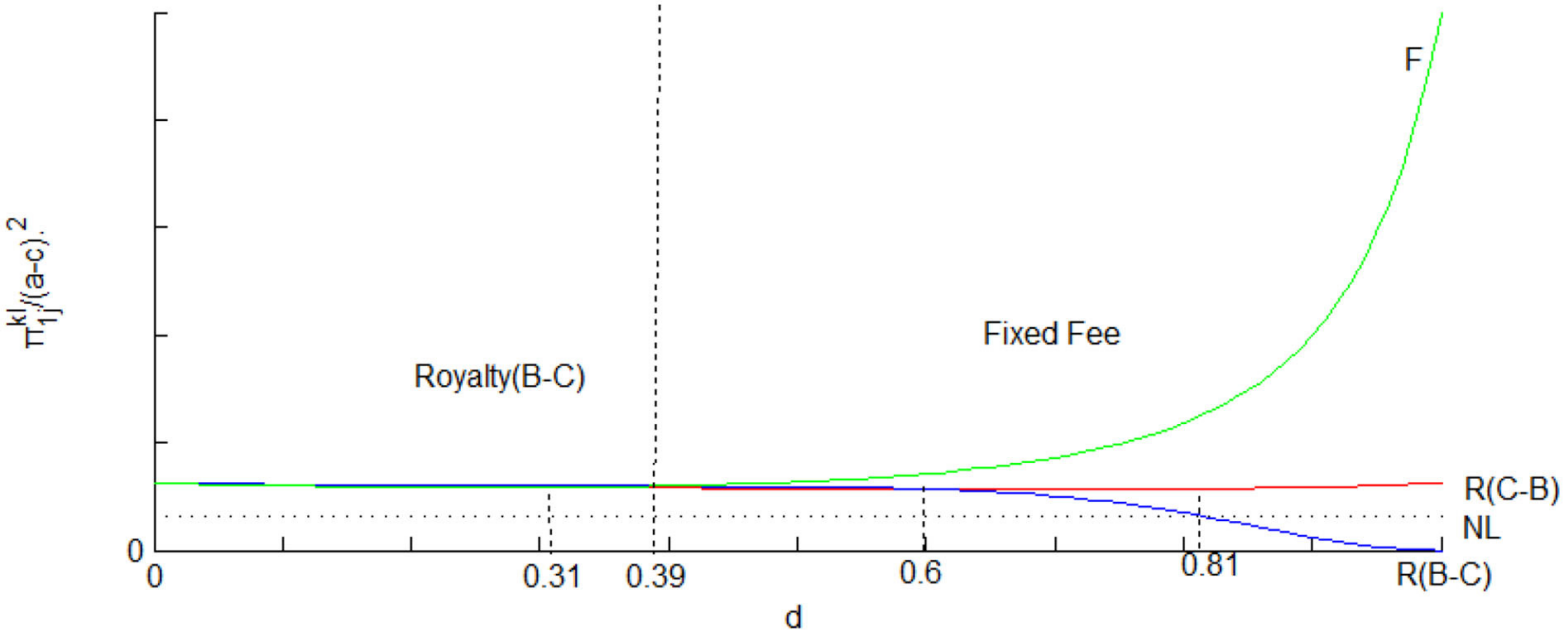
The profits of Firm 1 in different licensing methods under mixed competition.

As we can see in Figure 1, the profit of Firm 1 decreases as the degree of substitution increases. We note from (37) that an increase in the royalty rate reduces the equilibrium quantities of both the licensor and licensee, resulting in decreased revenue through licensing. The higher equilibrium price and lower charge from the supplier just do not offset this side effect. When the degree of substitution increases, the supplier has an incentive to reduce the wholesale price to keep the output to extract part of the resulting higher downstream profits. Having said that, the whole output in the downstream market falls off, which can be seen from the calculation. So the supplier might be better off without royalty licensing as the two products are distant substitutes under the Bertrand-Cournot competition.

And the responding consumer surplus and social welfare are:


$$\begin{array}{l} CS_{R}^{CB}=[a(q_{1}+q_{2})-\frac{q_{1}^{2}+2q_{1}q_{2}+q_{2}^{2}}{2}-p_{1}q_{1}-p_{2}q_{2}]\\ \;=\frac{\begin{array}{c} (972d^{12}+972d^{15}-7668d^{14}-6912d^{13}+26487d^{12}\\ +21006d^{11}-52659d^{10}-35460d^{9}\\ +66368d^{8}+36034d^{7}-54659d^{6}-\\ 22136d^{5}+28888d^{4}+7648d^{3}\\ -9008d^{2}-1152d+1280){\left(a-c\right)}^{2} \end{array}}{8{\left(4-3d^{2}\right)}^{2}{\left(18d^{6},-48d^{4}+45d^{2}-16\right)}^{2}}, \end{array}$$

(41) $$\begin{array}{l} W_{R}^{CB}\;=\;\pi_{1R}^{CB}\;+\;\pi_{2R}^{CB}\;+\;\pi_{SR}^{CB}\;+\;CS_{R}^{CB}. \end{array}$$

**Proposition 2b:** *Under a royalty licensing method in Bertrand-Cournot competition, the condition of consumers gets worse than the no-licensing scenario when* 0.22 ≤ *d* ≤ 0.76*. Consumer surplus under royalty licensing exceeds than under no licensing only when the degree of vertical product differentiation is either too large or too small*.

## Fixed-fee licensing

### Cournot-Bertrand competition

In the previous analysis, we considered the benefits of licensing under an arrangement that specified only a royalty rate in mixed competition. Licensing methods can also involve fixed fees. If Firm 1 sells its patent to Firm 2 using a fixed-fee contract, Firm 2 has to pay a fixed fee (denoted as F). After the fixed-fee licensing agreement is reached, the two firms decide their own optimal price or quantity under mixed competition. Assuming the supplier has already known their competition strategies, which is the basis of setting prices. The equilibrium under the fixed-fee licensing regime in the mixed competition is discussed below. The profit functions in Cournot-Bertrand are as follows:


(42) $$\begin{array}{l} \pi_{1F}^{CB}\;=\;\left[a\left(1\;-\;d\right)\;-\;\left(1\;-\;d^{2}\right)q_{1}\;+\;dp_{2}\right]q_{1}\;-\;w_{1}q_{1}\;+\;F,\\ \;\pi_{2F}^{CB}\;=\;\left(p_{2}\;-\;w_{2}\right)\left(a\;-\;p_{2}\;-\;dq_{1}\right)\;-\;F. \end{array}$$

Maximizing respective profit yields, the response function is as follows:


(43) $$\begin{array}{l} q_{1}=\frac{a\left(1\;-\;d\right)\;+\;dp_{2}-w_{1}}{2\left(1\;-\;d^{2}\right)}\text{ and }p_{2}=\frac{a-dq_{1}+w_{2}}{2}. \end{array}$$

We can obtain the optimal quantity and price by the intersection of (43):


(44) $$\begin{array}{l} q_{1}\;=\;\frac{2a\;-\;ad\;+\;dw_{2}\;-\;2w_{1}}{4\;-\;3d^{2}},\\ \;q_{2}\;=\;\frac{2a\;-\;ad^{2}\;-\;ad\;+\;dw_{1}\;-\;\left(2\;-\;d^{2}\right)w_{2}}{4\;-\;3d^{2}},\\ \;p_{2}\;=\;\frac{2a\;-\;ad^{2}\;-\;ad\;+\;dw_{1}\;+\;\left(2\;-\;2d^{2}\right)w_{2}}{4\;-\;3d^{2}}. \end{array}$$

The price and quantity levels are as in (19) with *r* = 0.

Substituting *q*_1_ and *q*_2_ with the profit function of the supplier yields the wholesale prices:


(45) $$\begin{array}{l} \underset{w_{1},w_{2}}{Max}\pi_{s}\;=\;\left(w_{1}\;-\;c\right)q_{1}\;+\;\left(w_{2}\;-\;c\right)q_{2}\\ \;w_{1}\;=\;\frac{a\;+\;c}{2}\text{ and }w_{2}\;=\;\frac{a\;+\;c}{2}. \end{array}$$

As can be seen, the supplier will charge the same to both firms and fixed-fee licensing cannot reduce the double marginalization problem of the supply chain. By routine calculation, the quantities and prices under fixed-fee licensing in the Cournot-Bertrand competition are:


(46) $$\begin{array}{l} q_{1F}^{CB}\;=\;\frac{\left(2\;-\;d\right)\left(a\;-\;c\right)}{2\left(4\;-\;3d^{2}\right)},\;q_{2F}^{CB}\;=\;\frac{\left(2\;-\;d\;-\;d^{2}\right)\left(a\;-\;c\right)}{2\left(4\;-\;3d^{2}\right)},\\ \;p_{1F}^{CB}\;=\;\frac{\left(6\;-\;d\;-\;5d^{2}\;+\;d^{3}\right)a\;+\;\left(2\;+\;d\;-\;d^{2}\;-\;d^{3}\right)c}{2\left(4\;-\;3d^{2}\right)},\\ \;p_{2F}^{CB}\;=\;\frac{\left(6\;-\;d\;-\;4d^{2}\right)a\;+\;\left(2\;-\;2d^{2}\;+\;d\right)c}{2\left(4\;-\;3d^{2}\right)}. \end{array}$$

Firm 1 as the licensor will maximize its payoff with the constraint $\pi_{2F}^{CB}\geq \pi_{2N}^{CB}$. As explained earlier, Firm 2 will accept the license even if it is indifferent to licensing or not, so the maximum license fee is solved by $\pi_{2F}^{CB}\;=\;\pi_{2N}^{CB}$ as follows:


(47) $$\begin{array}{l} F^{CB}\;=\;\frac{{\left(a\;-\;c\right)}^{2}{\left(a\;-\;d\;-\;d^{2}\right)}^{2}}{4{\left(4\;-\;3d^{2}\right)}^{2}}. \end{array}$$

A series of outcomes is apparent in this situation:


$$\begin{array}{l} \pi_{1F}^{CB}\;=\;\frac{\left(2d^{4}\;+\;2d^{3}\;-\;4d\;+\;8\right){\left(a\;-\;c\right)}^{2}}{4\left(4\;-\;3d^{2}\right)},\\ \;\pi_{SF}^{CB}\;=\;\frac{\left(4\;-\;2d\;-\;d^{2}\right){\left(a\;-\;c\right)}^{2}}{4\left(4\;-\;3d^{2}\right)}, \end{array}$$

$$\begin{array}{l} CS_{F}^{CB}\;=\;\frac{\left(4d^{4}\;+\;2d^{3}\;-\;14d^{2}\;-\;5d\;+\;14\right){\left(a\;-\;c\right)}^{2}}{8{\left(4\;-\;3d^{2}\right)}^{2}}, \end{array}$$

(48) $$\begin{array}{l} W_{F}^{BC}\;=\;\pi_{1F}^{BC}\;+\;\pi_{SF}^{BC}\;+\;CS_{F}^{BC}\;=\\ \;\frac{\left(4d^{4}\;+\;8d^{3}\;-\;21d^{2}\;-\;15d\;+\;26\right){\left(a\;-\;c\right)}^{2}}{4{\left(4\;-\;3d^{2}\right)}^{2}}. \end{array}$$

**Proposition 3:** *The licensor finds it more profitable to license via a fixed-fee contract under Cournot-Bertrand competition compared with no-licensing, and such an arrangement is good for the supplier, the consumer, and the whole society*.

The proposition means that $\pi_{1F}^{CB}>\pi_{1N}^{CB}$ is satisfied regardless of the value of *d*. The supplier charges the same price as (*a* + *c*)/2 to both firms under fixed-fee licensing because the two firms are equally strong. The whole downstream production exceeds that in the case of no licensing so that the supplier gets more profits. Since the goods are heterogeneous, the game between the supplier and the firms is not a perfect duopoly. Therefore, the statement that total duopoly profits are less than monopoly profits (Arya and Mittendorf, 2006) is not applicable in this situation. It can be observed that the licensor's total income including fixed fees and the revenue from products given in (48) is greater than that given in (5) or (12).

### Bertrand-Cournot competition

The profit functions of fixed-fee licensing in the Bertrand-Cournot competition are as follows:


(49) $$\begin{array}{l} \underset{p_{1}}{Max\pi_{1F}^{BC}}\;=\;\left(p_{1}\;-\;w_{1}\right)\left(a\;-\;p_{1}\;-\;dq_{2}\right)\;+\;F,\\ \;\underset{q_{2}}{Max\pi_{2F}^{BC}}=\left[a\left(1-d\right)-\left(1-d^{2}\right)q_{2}+dp_{1}-w_{2}\right]q_{2}-F. \end{array}$$

Maximizing respective profit yields the response function:


(50) $$\begin{array}{l} p_{1}=\frac{a-dq_{2}+w_{1}}{2}\text{ and }q_{2}=\frac{a\left(1-d\right)\;+\;dp_{1}-w_{2}}{2\left(1-d^{2}\right)}. \end{array}$$

Then the supplier sets prices for the two firms to maximize their payoff and realizes the outcomes:


$$\begin{array}{l} \underset{w_{1},w_{2}}{Max}\pi_{s}=\left(w_{1}-c\right)q_{1}+\left(w_{2}-c\right)q_{2}. \end{array}$$

(51) $$\begin{array}{l} w_{1}\;=\;\frac{a\;+\;c}{2}\text{ and }w_{2}\;=\;\frac{a\;+\;c}{2}. \end{array}$$

Substituting (50) and (51) with (49), we get the quantities and prices:


$$\begin{array}{l} q_{1F}^{BC}\;=\;\frac{\left(2\;-\;d\;-\;d^{2}\right)\left(a\;-\;c\right)}{2\left(4\;-\;3d^{2}\right)},\\ \;q_{2F}^{BC}\;=\;\frac{\left(2\;-\;d\right)\left(a\;-\;c\right)}{2\left(4\;-\;3d^{2}\right)}, \end{array}$$

$$\begin{array}{l} p_{1F}^{BC}\;=\;\frac{\left(6\;-\;d\;-\;4d^{2}\right)a\;+\;\left(2\;-\;2d^{2}\;+\;d\right)c}{2\left(4\;-\;3d^{2}\right)}, \end{array}$$

(52) $$\begin{array}{l} p_{2F}^{BC}\;=\;\frac{\left(6-d-5d^{2}+d^{3}\right)a+\left(2+d-d^{2}-d^{3}\right)c}{2\left(4-3d^{2}\right)}. \end{array}$$

Interestingly the price and quantity of the two firms under two kinds of mixed competition are interchanged. Firm 1 as the licensor will maximize its payoff with the constraint $\pi_{2F}^{BC}\geq \pi_{2N}^{BC}$. Making $\pi_{2F}^{BC}\;=\;\pi_{2N}^{BC}$, we obtain the fixed fee:


(53) $$\begin{array}{l} F^{BC}\;=\;\frac{{\left(a\;-\;c\right)}^{2}{\left(a\;-\;d\;-\;d^{2}\right)}^{2}}{4{\left(4\;-\;3d^{2}\right)}^{2}}. \end{array}$$

So we can get the equilibrium profits and consumer surplus and welfare as follows, which are also the same as competition in Cournot-Bertrand.


$$\begin{array}{l} \pi_{CB}^{1F}\;=\;\frac{\left(2d^{4}\;+\;2d^{3}\;-\;4d^{2}\;-\;4d\;+\;8\right){\left(a\;-\;c\right)}^{2}}{4{\left(4\;-\;3d^{2}\right)}^{2}},\\ \;\pi_{SF}^{BC}\;=\;\frac{\left(4\;-\;2d\;-\;d^{2}\right){\left(a\;-\;c\right)}^{2}}{4{\left(4\;-\;3d^{2}\right)}^{\;}}, \end{array}$$

$$\begin{array}{l} CS_{F}^{BC}\;=\;\frac{\left(4d^{4}\;+\;2d^{3}\;-\;14d^{2}\;-\;5d\;+\;14\right){\left(a\;-\;c\right)}^{2}}{8{\left(4\;-\;3d^{2}\right)}^{2}}, \end{array}$$

(54) $$\begin{array}{l} W_{F}^{BC}\;=\;\pi_{1F}^{BC}\;+\;\pi_{SF}^{BC}\;+\;CS_{F}^{BC}\;=\\ \;\frac{\left(4d^{4}\;+\;8d^{3}\;-\;21d^{2}\;-\;15d\;+\;26\right){\left(a\;-\;c\right)}^{2}}{4{\left(4\;-\;3d^{2}\right)}^{2}}. \end{array}$$

**Proposition 4a:** *The licensor finds it more profitable to license via a fixed-fee contract under Bertrand-Cournot competition compared with no-licensing, and such an arrangement also benefits the supplier, the consumer, and the whole society*.

**Proposition 4b:** *The type of mixed competition, Cournot-Bertrand or Bertrand-Cournot, does not make any difference to the participants in the market when fixed-fee licensing occurs*.

## Comparative analysis

This paper discussed the equilibrium quantities, prices, profits of three parties, consumer surplus, and social welfare under no-licensing, royalty licensing, and fixed-fee licensing in mixed competition. To accurately analyze the optimal licensing regime, the implications of the two licensing methods under the Cournot-Bertrand and Bertrand-Cournot competitions were compared.

Figure 1 depicts the curves $\pi_{1j}^{kl}$, from which we can tell the profits of Firm 1 in different situations at the same time. As we can see, Firm 1's total income in the fixed-fee licensing option is smaller than in the royalty licensing option under the Cournot-Bertrand competition when 0 < *d* < 0.31 and even smaller than in the royalty licensing option under the Bertrand-Cournot competition when 0 < *d* < 0.39. Meantime, Firm 1 is better off under Bertrand-Cournot than Cournot-Bertrand competition. Therefore, Firm 1 would prefer royalty to fixed-fee licensing under both two types of mixed competition when the degree of substitution is limited to 0 < *d* < 0.39. Furthermore, Firm 1 will choose the fixed-fee licensing if 0.39 < *d* < 1. We get the following conclusion from the above discussion.

**Proposition 5:** *Relative to the Cournot-Bertrand competition, the licensor prefers the royalty to the fixed fee under the Bertrand-Cournot competition if the degree of substitution is no more than 0.39. If the degree of substitution is higher than 0.39 Firm 1 would choose fixed-fee licensing no matter under what type of mixed competition*.

The intuition for proposition 5 is as follows. Firm 1 as a dominant party in the duopoly market chooses price as the decision variable to gain more market share while alternatively if quantity is chosen as the decision variable, Firm 1 can gain significantly from royalty licensing. Hence, Firm 1 prefers Bertrand-Cournot to Cournot-Bertrand competition when royalty licensing occurs. However, the standard result according to Singh and Vives (1984) is that the licensor's profit under Cournot-Bertrand competition is higher than that under Bertrand-Cournot competition with other things constant. The reversal from this is driven by the upstream supplier's stronger incentives to increase the competitiveness of the downstream firms under Cournot-Bertrand competition than Bertrand-Cournot competition. That leads to lower wholesale prices for Firm 1 and thus higher profit. As the degree of substitution gets larger, with the severity of competition becoming smaller, the licensor has a lower incentive to use the royalty to alleviate market competition; instead, the licensor prefers a fixed fee to extract more licensing revenue.

**Figure 2:**
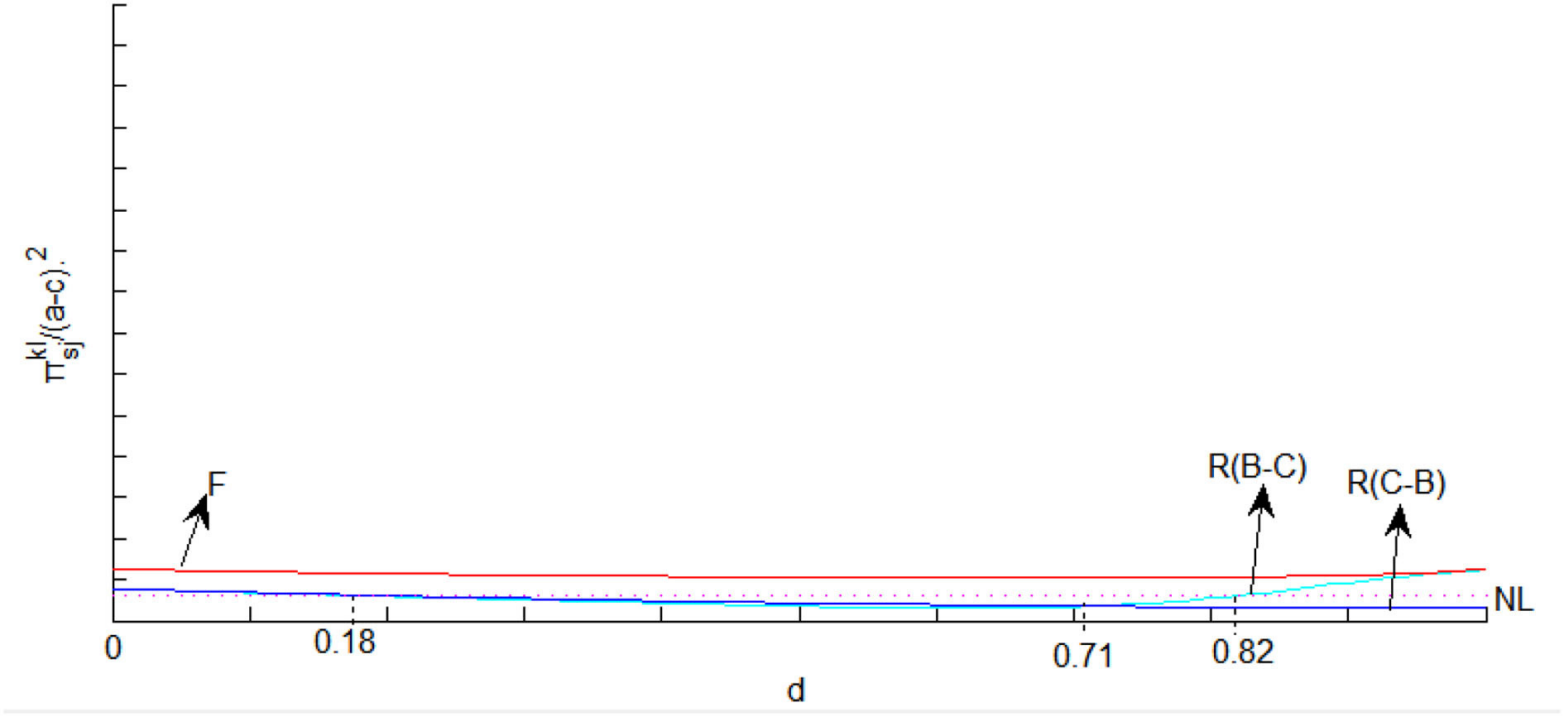
The profits of the supplier in different licensing methods under mixed competition.

**Figure 3:**
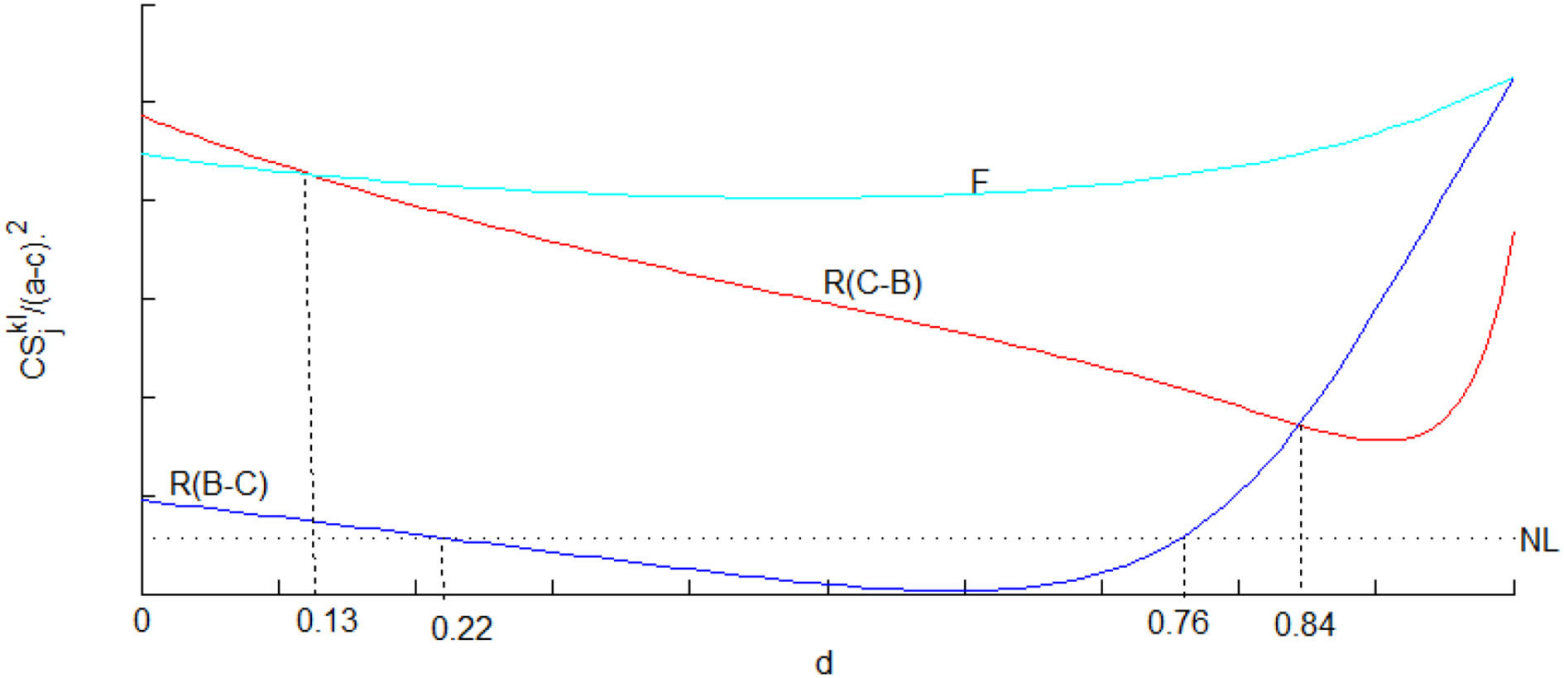
Consumer surplus in different licensing methods under mixed competition.

**Figure 4:**
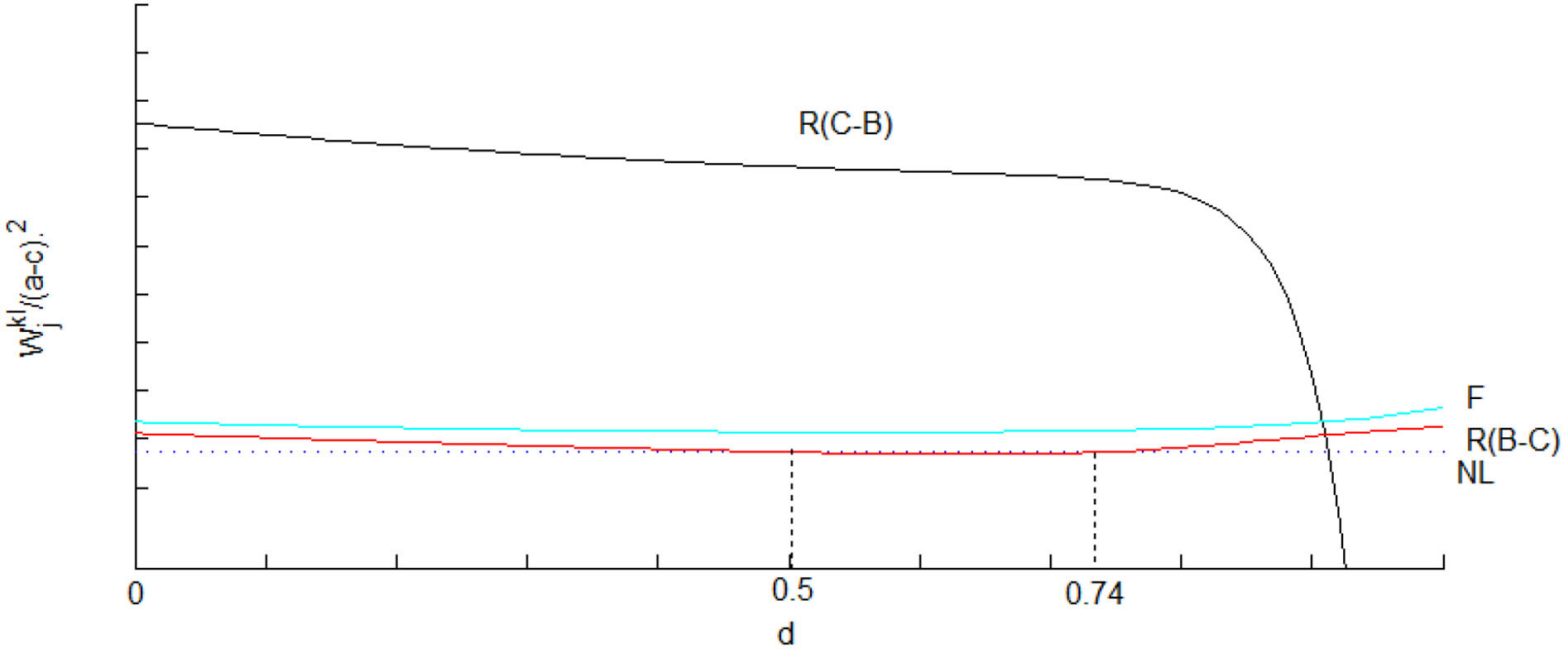
Society welfare in different licensing methods under mixed competition.

We further use Figures 2–4 to analyze the implications of licensing strategies under different mixed competitions from other parties' perspectives. As shown in Figure 2, fixed-fee licensing is beneficial for the supplier, the reason being that the downstream output and the wholesale price in fixed-fee licensing are greater than those in royalty licensing. While there is not much difference between the two types of mixed competition when royalty licensing occurs, which is the last choice of the supplier since the profits are less than that in no licensing case if the degree of substitution exceeds 0.18. In contrast to the assertion by Wang (2002) that fixed-fee licensing is always superior for the consumer, we find the exception that royalty licensing under Cournot-Bertrand competition is the best choice for consumers if the degree of substitution is small enough. The whole society consistently benefits from Firm 1's licensing behavior even though royalty licensing under Cournot-Bertrand competition is superior if the degree of substitution is not high enough.

Table 1 presents the summary of all propositions drawn from this study.

**Table 1:** The summary of propositions.

**Proposition**	**Licensing contract**	**Competition mode**	**Results**
Proposition 1	Royalty licensing	Cournot-Bertrand competition	(1) The patentor is strictly profitable; (2) Benefits consumers and society; (3) Hurts the supplier.
Proposition 2	Royalty licensing	Bertrand-Cournot competition	(1) The patentor is profitable when 0*id* ≤ 0.81; (2) Benefits consumers when 0 < *d* < 0.22 or 0.76 < *d* < 1.
Proposition 3	Fixed-fee licensing	Cournot-Bertrand competition	Benefits the licensor, consumers, society, and supplier.
Proposition 4	Fixed-fee licensing	Bertrand-Cournot competition	Benefits the licensor, consumers, society, and supplier
Proposition 5	(1) The licensor prefers the royalty to the fixed fee under the Bertrand-Cournot competition if *d* ≤ 0.39 relative to the Cournot-Bertrand competition; (2) The fixed-fee option is preferred by the licensor no matter the competition mode if *d*> 0.39.		

## Conclusion

In this paper, we proposed a mixed competition model, assuming that there are two downstream firms and one upstream supplier in a differentiated duopoly market. One of the firms has an innovative technology and the other is a potential licensee, and we try to analyze the optimal licensing strategy for the licensor.

We show that relative to the Cournot-Bertrand competition, the licensor prefers the royalty to the fixed fee under the Bertrand-Cournot competition if the degree of substitution is no more than 0.39 and would transfer the technology *via* royalty licensing rather than fixed-fee licensing. This conclusion is different from Chang et al. (2015), which show that under Cournot–Bertrand and Bertrand–Cournot competitions the licensor prefers the fixed fee to a royalty when the degree of innovation gets larger. If the degree of substitution is higher than 0.39, Firm 1 prefers to choose fixed-fee licensing no matter under what type of mixed competition. Besides, the type of mixed competition, Cournot-Bertrand or Bertrand-Cournot, does not make any difference to the participants in the market when fixed-fee licensing occurs. We also investigated the optimal licensing strategies from other participants' perspectives. A major conclusion was that fixed-fee licensing is not always the best choice for consumers as long as the degree of substitution is sufficiently small.

In today's economy, many firms outsource their inputs to external suppliers and consider it an essential part of their overall business strategy. Suppliers have a significant influence on the decision of licensing and since they set the price for the final product, firms have to consider the behavior of suppliers to maximize their profit and improve the efficiency of the supply chain. This paper also shows that royalty licensing can reduce the double marginalization problem of the supply chain, while fixed-fee licensing cannot.

Further research may extend our study as follows: First, this paper examined the fixed-fee licensing and the royalty licensing in the case where there is only one upstream supplier without identifying the optimal number of licenses. However, in recent years, particularly since the outbreak of the coronavirus, supply chain disruption or supply chain uncertainty is a significant factor that affects technology licensing. Therefore, supply chain uncertainty should be considered in future research. Second, this paper did not incorporate more complex licensing methods such as two-part tariff licensing and the degree of innovation, which may be an interesting topic for further research.

## Data availability statement

The original contributions presented in the study are included in the article/supplementary material, further inquiries can be directed to the corresponding author.

## Author contributions

HZ performed study concept and design. YZ performed development of methodology and writing of the paper. MZ provided financial support. All authors read and approved the final paper.

## Funding

This research was supported in part by the Natural Science Foundation of Guangdong Province (Grant No. 2021A1515011569) and the 13th Five-Year Plan Foundation of Philosophy and Social Sciences of Guangdong Province (Grant No. GD20CGL55).

## Conflict of interest

The authors declare that the research was conducted in the absence of any commercial or financial relationships that could be construed as a potential conflict of interest.

## Publisher's note

All claims expressed in this article are solely those of the authors and do not necessarily represent those of their affiliated organizations, or those of the publisher, the editors and the reviewers. Any product that may be evaluated in this article, or claim that may be made by its manufacturer, is not guaranteed or endorsed by the publisher.
